# Blood pressure dynamics during home blood pressure monitoring with a digital blood pressure coach—a prospective analysis of individual user data

**DOI:** 10.3389/fcvm.2023.1115987

**Published:** 2023-04-05

**Authors:** Christian Beger, Dominik Rüegger, Anna Lenz, Steffen Wagner, Herrmann Haller, Kai Martin Schmidt-Ott, Dirk Volland, Florian P. Limbourg

**Affiliations:** ^1^ Vascular Medicine Research, Department of Nephrology and Hypertension, Hannover Medical School, Hannover, Germany; ^2^ Department of Nephrology and Hypertension, Hannover Medical School, Hannover, Germany; ^3^ Pathmate Technologies GmbH, Mannheim, Germany; ^4^ Department II (Mathematics, Physics and Chemistry), Berliner Hochschule für Technik, Berlin, Germany; ^5^ INWT Statistics GmbH, Berlin, Germany

**Keywords:** hypertension, home blood pressure monitoring, app, eHealth, blood pressure

## Abstract

**Introduction:**

Self-monitoring of blood pressure at home is a better predictor of prognosis and recommended in hypertension guidelines. However, the influence of baseline blood pressure category and measurement schedule on BP values during a period of home blood pressure monitoring (HBPM) are still poorly defined, particularly when used in conjunction with a digital application.

**Methods:**

We analysed temporal BP changes and performed BP classification tracking in users with self-reported hypertension performing HBPM with a digital and interactive blood pressure coach.

**Results:**

Of 3175 users who enrolled in HBPM, 74.1% completed the first measurement period. Overall, mean systolic BP dropped significantly after the first day, but stratification by BP category demonstrated that initial category influenced BP course. BP classification tracking revealed that time to reach final BP category was dependent on baseline category, with users in categories high normal and grade 1 hypertension requiring more days to decrease BP class volatility and to reach their definitive BP class. This was driven by an intense switching between directly neighbouring categories until the middle phase of the HBPM period, while more distant class switching occurred less often and only early on. Overall, >90% of users maintained their category by day 5. Omitting the first day from analysis lead to therapeutically relevant reclassification in 3.8% of users. Users who completed at least two HBPM periods (*n* = 864) showed a mean SBP/DBP decrease of 2.6/1.6 mmHg, which improved hypertension control from 55.6% to 68.1%.

**Conclusion:**

The optimal length of HBPM period depends on BP category. HBPM with a digital coach is associated with a reduction in average BP and improvement in BP control.

## 1. Introduction

Hypertension is the most important risk factor for cardiovascular diseases or premature death (1), yet hypertension control at the population level and in treated patients remains suboptimal (2, 3). For decades, screening and management of hypertension were primarily based on office blood pressure [OBP (4)]. Alternatively, out-of-office measurements such as home blood pressure monitoring (HBPM) can be used for disease management. HBPM is based on self-measurements at home according to a structured protocol (5–7). Numerous studies have shown that HBPM in comparison with OBP has the potential to improve adherence and hypertension (HTN)-control. In addition, blood pressure (BP) readings obtained at home according to a structured protocol have significantly higher prognostic relevance than OBP values (4, 8–11). Thus, current guidelines recommend HBPM for the management of patients with hypertension (5, 7).

However, inaccurate BP-measurements or unstructured schedules may limit the clinical benefits of HBPM. Adequate HBPM requires appropriate training, constant motivation and patient guidance. With limited resources, practical implementation in everyday clinical practice can be challenging (12). In this context digital solutions may offer new approaches to support medical treatment processes. The possible opportunities of digital HBPM interventions go far beyond simple BP value tracking—applications could in particular support patient empowerment and promote BP self-management (13, 14).

Therefore, we aimed to analyse usage behaviour, user adherence and possible clinical effects of a guideline-compliant HBPM-protocol implemented by an interactive chatbot (“digital coach”). The coaching app guides users to measure and document their blood pressure correctly and regularly in accordance with current guideline recommendations and provides an assessment based on home BP categories. However, current guidelines differ in terms of the proposed HBPM schedule (e.g., required measurement days and timing, relevance of the first day) (5, 7, 15). We therefore investigated the influence of measurement duration and baseline BP category on BP-categorization in a real-world setting, when a digital coach implements HBPM.

## 2. Material and methods

### 2.1. Study design

This study is a prospective analysis of real-world user data from a digital BP-coach (Manoa app) in the period from April 2020 (release of the Manoa app) to November 2021 in Germany. The app was freely available in Germany during this period in all major app stores (iOS, Android). The Department of Nephrology and Hypertension, Hanover Medical School initiated this analysis. It was approved by the local ethics committee (9033_B0_K_2020). Users gave their informed consent that their data may be evaluated for scientific purposes.

### 2.2. Coaching app

Manoa is a conversational smartphone app which collects user information and provides coaching and support to promote hypertension self-management. It also covers topics like healthy lifestyle (e.g., healthy diet, physical activity) and medication adherence. The app was developed in collaboration with Hannover Medical School (MHH).

Core element is the blood pressure diary. The digital coach provides individual feedback on the user's blood pressure level, based on the results from a structured HBPM protocol. Encouragement by the digital Coach “Manoa” (chatbot), reminders, and tracking are used to promote home monitoring. The app motivates users to measure their blood pressure twice a day (every morning and evening) for 6 days (HBPM period). After completion of the first HBPM period, the user is asked and reminded periodically (every 4 weeks) to monitor his or her blood pressure. The app also allows the user to reschedule the HBPM period. Users can record their blood pressure manually, scan the result *via* the smartphone camera or import the blood pressure values directly from a compatible measurement device. For the analysis, the first HBPM period (baseline) was considered as successfully completed if the user had taken 2 measurements per day (in the morning and in the evening) on at least 6 days within a period of 6–14 days. For the following HBPM periods, 3 complete measurement days (each with one measurement in the morning and in the evening) within 7 days were sufficient.

During the first HBPM period, the user receives relevant information (e.g., about the correct blood pressure measurement at home) through puzzles, articles or videos, for example. After completing an HBPM measurement period, the user receives evaluative feedback and further recommendations based on his blood pressure values. In addition, the app offers a visualisation of the blood pressure values in a diary. This diary can be exported and shared with the doctor.

### 2.3. Participants and diagnostic principles

The participants downloaded the app voluntarily and self-motivated. Persons under 18 and pregnant women were excluded from app usage. The analysis is based on all users with self-reported diagnosis of hypertension who started a first measurement week (*n* = 3175). Depending on the analysis, subgroups were defined as indicated.

Hypertension was diagnosed according to current guideline recommendations (5). Users were classified in normal BP, high normal BP, grade I hypertension and grade II hypertension according to outcome-driven thresholds, as previously published (7, 16, 17). Home BP was analysed at baseline and 8–16 weeks after successful completion of the first HBPM period (follow up).

### 2.4. Statistical analysis

The analyses were performed with the open source statistical software R or with GraphPad Prism 9.0. Characteristics of users were summarised as numbers and percentage for categorical variables and mean and SD for continuous variables. The change in the blood pressure level (mean) was analysed with a paired t-test. Differences in categorical variables were compared through a *χ*2 test. A generalized additive mixed model (GAMM) that considers the repeated measures and a potential non-linear relationship between BP values and day of measurement was applied in the analysis using the R package *mgcv* (v1.8.40) (18, 19). One result of the applied GAMM models is the reported effective degrees of freedom (EDF) which is a measure of the extent of observed non-linearity, i.e., the curvature of the smoothing spline function. A value of EDF = 1 indicates a linear relationship between BP and time, whereas EDF close to 2 indicates a parabolic curve shape. The identified relationships along with the 95% confidence intervals are plotted and the discussion of non-linearities is based on the plots.

Pearson correlation test was performed to evaluate the relationship between change in systolic BP (follow-up—baseline) and systolic BP at baseline. Piecewise linear regression was used to test if baseline systolic BP predicted change (follow up—baseline) in systolic BP. Cohen's kappa was computed to assess the agreement between different HBPM protocols in classifying hypertension.

## 3. Results

### 3.1. Baseline characteristics

From April 2020 to November 2021, datasets from 3,175 app-users with self-reported arterial hypertension were analysed, while the app was freely available in all major app stores in Germany. The baseline characteristics are summarised in Table 1. The mean age (SD) of the participants was 61.6 (10.4) years. 55.6% of the users were older than 60 years and 1,527 (48.1%) of them were female. In the subgroup of users who reported their last OBP (*n* = 2666), the average reported OBP (SD) was 138.7 (14.4) /83.9 (9.7) mmHg, which is in the controlled range for systolic BP (SBP) and diastolic BP (DBP) (<140/90 mm Hg). However, 54.1% of users had at least one OBP value (systolic or diastolic) which classified them as uncontrolled. We also queried previous HBPM experience of users at the beginning of the coaching program (*n* = 3983). 51.0% of users had performed HBPM regularly, while 47.8% of users had performed HBPM sporadically and 1.3% of users had never performed HBPM (Table 1).

**Table 1:** Baseline characteristics of users with self-reported hypertension.

Characteristics	Total (*n* = 3175)	Female (*n* = 1527)	Male (*n* = 1648)
Age, years	61.6 ± 10.4	60.1 ± 9.9	63.0 ± 10.8
Age groups (%)			
18–30	21 (0.7)	12 (0.8)	9 (0.6)
31–40	77 (2.4)	34 (2.2)	43 (2.6)
41–50	320 (10.1)	181 (11.9)	139 (8.4)
51–60	992 (31.2)	548 (36.0)	444 (27.0)
61–270	1,138 (35.8)	533 (34.9)	605 (36.7)
>71	627 (19.8)	219 (14.3)	408 (24.8)
BMI	28.1 ± 5.2	28.3 ± 6.0	27.9 ± 4.3
Systolic OBP, mmHg	138.7 ± 14.4 (*n* = 2666)	139.9 ± 15.1 (*n* = 1297)	138.1 ± 13.7 (*n* = 1369)
Diastolic OBP, mmHg	83.9 ± 9.7 (*n* = 2666)	84.7 ± 10.2 (*n* = 1297)	83.2 ± 9.3 (*n* = 1396)
sOBP uncontrolled %	49.0	49.7	48.4
dOBP uncontrolled %	30.1	32.9	27.4
OBP uncontrolled %	54.1	55.3	53.0
Medication (%)*	2,825 (89.0)	1,356 (88.9)	1,469 (89.1)
User experience (HBPM)	Frequent	Sporadic	Never
Participants (%)	2,031 (51.0)	1,902 (47.8)	50 (1.3)

BMI, body mass index; HBPM Home blood pressure monitoring; OBP office blood pressure; sOBP systolic office blood pressure; dOBP diastolic office blood pressure. Age, BMI, systolic and diastolic OBP values are expressed as mean ± SD. Uncontrolled OBP: systolic BP ≥ 140 mm Hg and/or diastolic BP ≥ 90 mm Hg.

* Reported antihypertensive medication status (*n* = 3174). If users have started an HBPM period, the last status before the first HBPM period is reported. Otherwise, the first documented status was analysed.

### 3.2. User adherence during baseline HBPM interval

**Figure 1:**
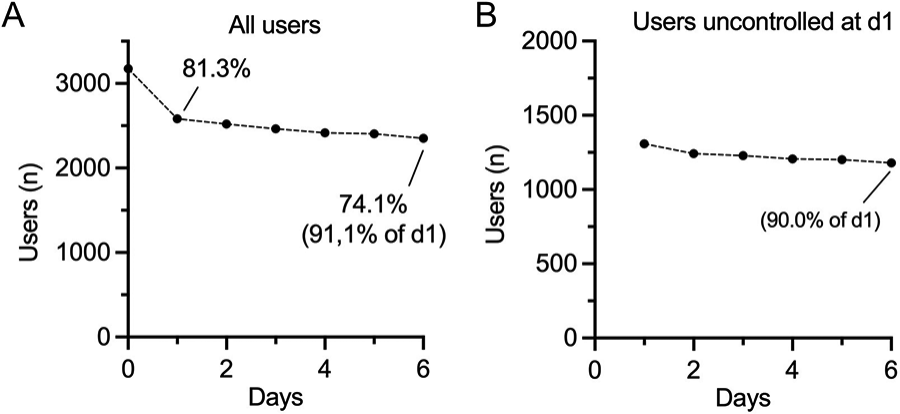
User numbers during baseline HBPM interval. (**A**) Number of users with self-reported diagnosis of hypertension who started the first HBPM interval (*n* = 3175). Percentages are given in brackets. (**B**) Number of users with a self-reported diagnosis of hypertension who have started a first HBPM interval and are uncontrolled on the first day of measurement (*n* = 1309). Percentages for day 2 and 6 are given in brackets.

Current guidelines recommend the completion of a structured HBPM protocol to evaluate ambulatory blood pressure values (5). Therefore, we first analysed the rate of self-reported hypertensive users who successfully completed the first HBPM period with the digital coach (completion of at least 6 BP measurement days). Of 3,175 users with self-reported hypertension who enrolled in HBPM, 81.3% (2582) completed the first day. During the ensuing HBPM interval, there was a steady decline in active users by 1%–2% per day, resulting in an overall successful completion rate of 74.1% (2,352 out of 3175) in this strictly self-motivated and app-coached setting (Figure 1A). Thus, the majority of users was lost during the HBPM initiation process (18.7%), while a smaller fraction of users (7.2%) was lost during the actual measurement period. 91.1% of users (2,352 out of 2582) who started the first measurement day completed the entire HBPM period (Figure 1A). In the subgroup of users who had uncontrolled hypertension on the first day of the HBPM period, the same relative change of user numbers was observed in the subsequent measurement days (Figure 1B). The different age groups did not differ in their usage behaviour (Supplementary Figure S1).

### 3.3. BP course and categories during first HBPM period

The digital coach supports users in implementing and documenting a structured HBPM-protocol. However, despite the widespread use of HBPM, the exact number of days required for an adequate classification is still matter of debate and accuracy in relation to BP categories is unclear. A recent meta-analysis concluded that a three-day protocol is sufficient in most cases, but should be extended to seven days in case of doubt (20). Furthermore, it is currently unclear how baseline or final (definitive) BP category influences BP course and HBPM protocol requirements. We therefore analysed temporal BP patterns and BP course stratified by BP categories of participants of the first HBPM period.

**Figure 2:**
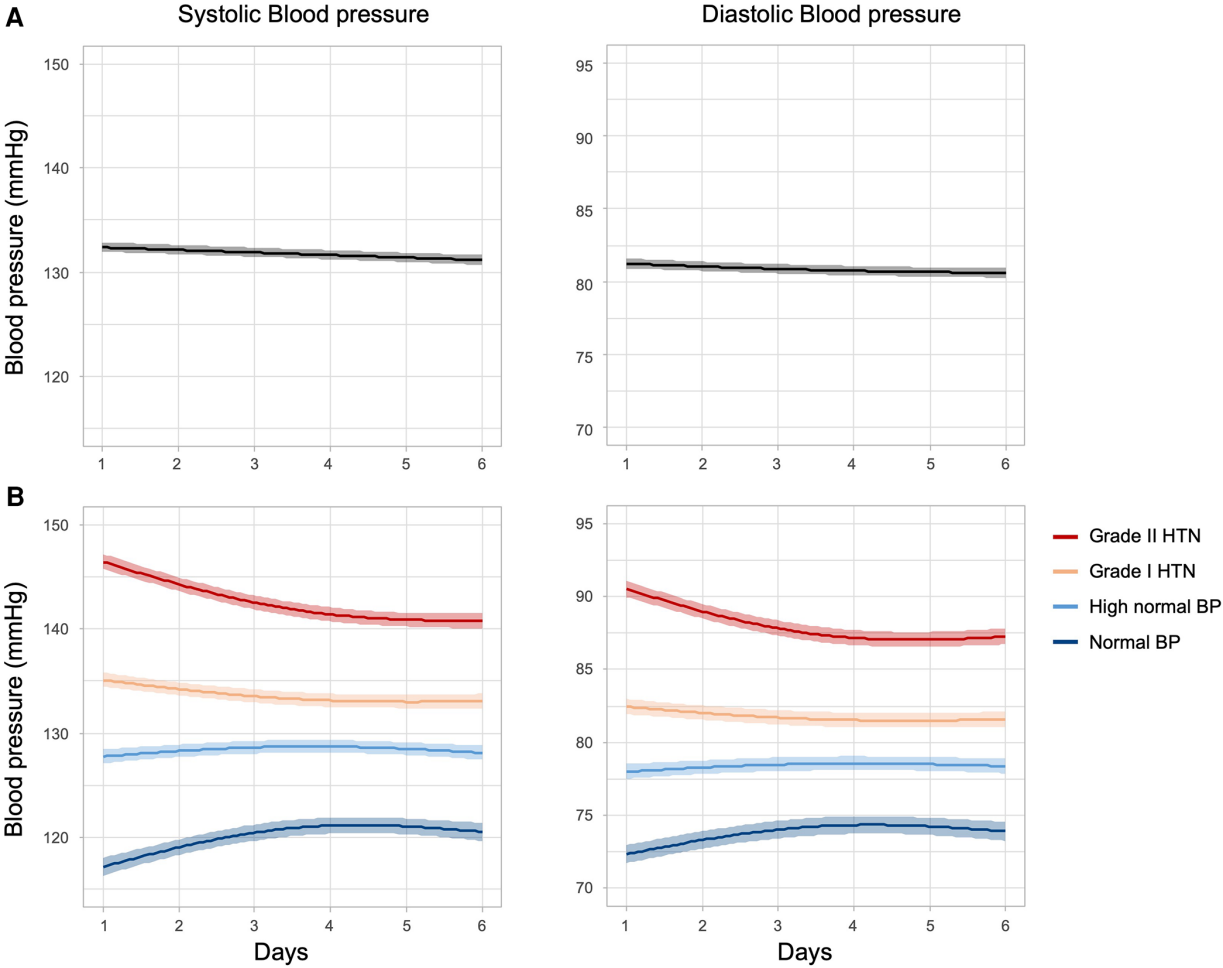
Home blood pressure values in the first HBPM interval. Shown are plots of predicted smoothing spline functions of time (solid lines) with 95% confidence bands (matt surrounding). (**A**) Course of systolic and diastolic blood pressure values of users with self-reported hypertension in the first HBPM period. (**B**) Course of SBP and DBP stratified by initial (day 1) BP category. Smooth functions for systolic BP as a function of time show significant non-linear relationships (normal BP: EDF = 1.98, *p* < 2 × 10^−16^; high normal BP: EDF = 1.91, *p* = 5.4 × 10^−3^; grade I HTN: EDF = 1.91, *p* < 2 × 10^−16^; grade II HTN: EDF = 1.98, *p* < 2 × 10^−16^) as well as the diastolic BPs (normal BP: EDF = 1.97, *p* < 2 × 10^−16^; high normal BP: EDF = 1.85, *p* = 1.8 × 10^−2^; grade I HTN: EDF = 1.88, *p* = 1.6 × 10^−5^; grade II HTN: EDF = 1.99, *p* < 2 × 10^−16^). The observed EDFs identical or close to 2 indicate a parabolic relationship in all scenarios although the sign of the effects differs, i.e. BP increase as well as BP decrease over time is observed. Analyses are based on data from users with self-reported hypertension who enrolled in HBPM and completed the first day (*n* = 2582).

Across all participants, mean SBP (SD) was highest after the first measurement day [132.6 (13.0)]. During the following measurement days, systolic and diastolic blood pressure decreased significantly over time. Using generalized additive mixed modelling (GAMM), systolic BP decreased in a linear fashing with a slope of -0.25(3) mmHg/day (EDF = 1, *p* = 2.14 × 10^−14^), whereas diastolic BP decreased non-linearly (EDF = 1.71, *p* = 6.9 × 10^−8^) with changes being greatest between day 1 and day 2 (Figure 2A). A linear approximation over the whole time period resulted in a slope of −0.13(2) mmHg/day (*p* = 2.8 × 10^−10^).

To understand BP volatility in more detail, we next stratified users according to their initial hypertension category (based on day 1 BP). In this analysis the significant changes (details see caption of Figure 2) in blood pressure over time were most pronounced in the categories at the respective ends of the diagnostic spectrum, with the normotensive user group even showing an increase in blood pressure over time. In users categorised as high normal and grade I hypertension only minor changes were detected over time (Figure 2B). Although the sign of the temporal BP patterns across the BP categories differs (increasing vs. decreasing BP), a stable BP level was reached at around day 4 of measurement.

**Figure 3:**
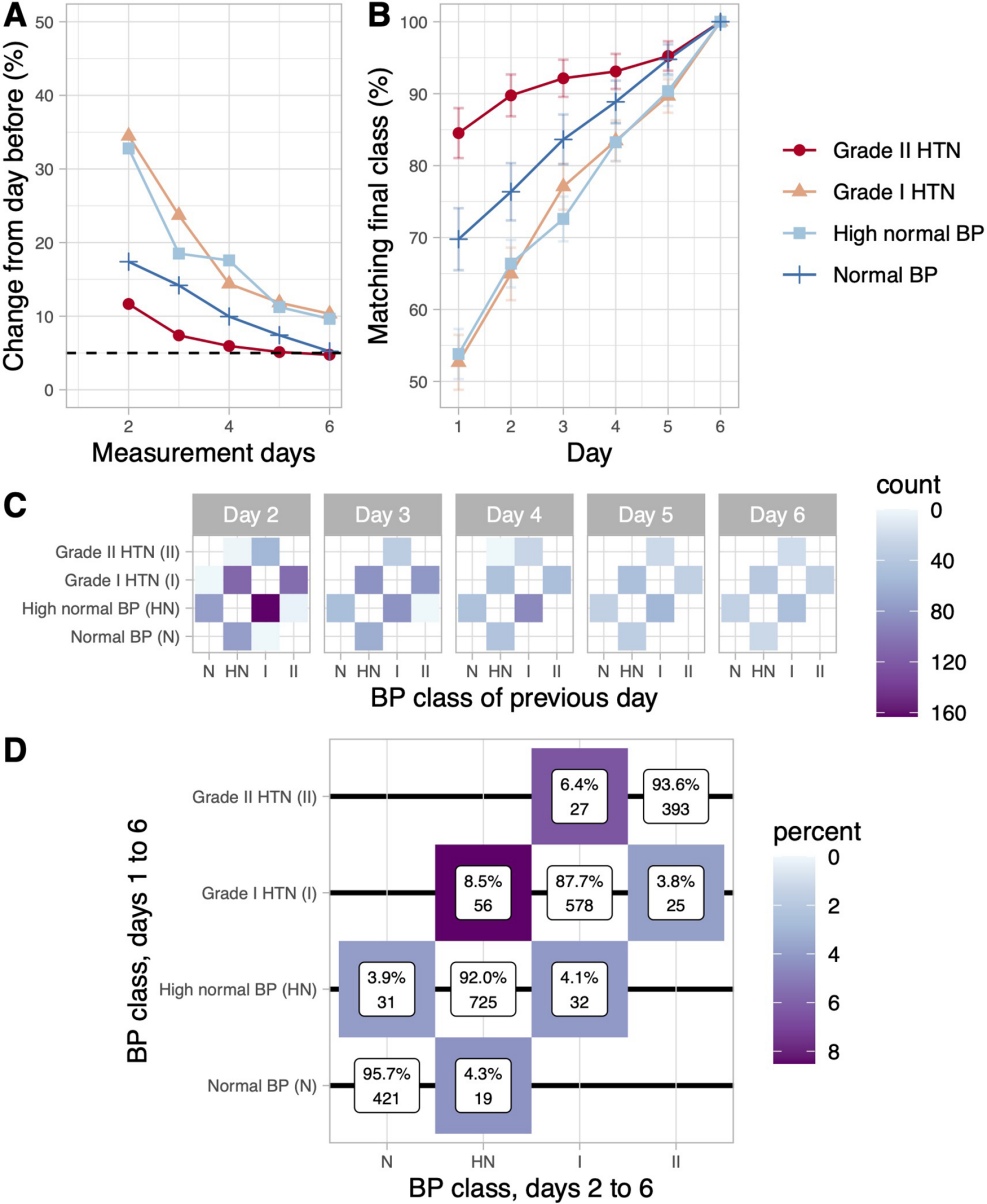
Blood pressure classification tracking during the HBPM protocol. (**A**) Fraction of users who were reclassified compared to the previous day; classification is always based on the cumulative BP mean of consecutive measurement days. (**B**) Percentage of users who already matched their final BP-category. Classification is based on the cumulative BP-mean of day 1–6. (**C**) Number of patients who changed their classification compared to the classification from the previous day. (**D**) Change in blood pressure categories after exclusion of day 1. The crosstab shows how users (grouped by category based on day 1–6 mean, vertical) are classified according to a day 2–6 protocol. (**A–D**) Analyses are based on data obtained from users with self-reported hypertension who completed the first HBPM -period (*n* = 2307).

Various studies have shown that an increase in measurement days is associated with better reproducibility and accuracy (17, 20, 21). Since correct BP-classification (e.g., normotension, grade I HTN, grade II HTN) informs prognosis and therapeutic decisions, we examined how the increase in measurement days affects user BP-classification. Overall, the proportion of patients to be reclassified compared to the previous day (based on the cumulative mean) decreased with an increasing number of consecutive measurement days. On day 2, agreement with the previous day was 74.3%, while on day 5 > 90% of users maintained their classification (Table 2). This finding is based on a stabilisation of the mean BP-value over the course of the HBPM period, which can be attributed to the increase in the measured values. However, BP reclassification over time and time to reach definitive BP class was not uniform. At the extremes of the diagnostic spectrum (normotension, grade II HTN), reclassification over consecutive measurement days occurred less frequently and a high proportion of users matched their final category early on (defined by the mean of a complete HBPM period, Figures 3A,B). Of note, 89.8% of users with BP readings in the grade II range were correctly classified at day 2. In contrast, users in BP categories high normal and grade 1 hypertension required more days to decrease BP class volatility and reach their definitive BP class, with 90.4% and 89.8% correctly classified, respectively, at or after day 5 (Figure 3B). Thus, a higher number of measurement days is required for adequate classification when BP values are close to the therapeutic threshold. However, accuracy was improved in all categories when measurements until day 6 were integrated.

**Table 2:** Cumulative mean of consecutive measurement days for BP-classification.

	Cumulative home measurement days					
*Category*	1	1–2	1–3	1–4	1–5	1–6
Normal	431 (NA)	431 (17.4)	444 (14.2)	442 (10.0)	445 (7.4)	440 (5.2)
High normal	681 (NA)	735 (32.8)	724 (18.5)	768 (17.6)	774 (11.2)	788 (9.6)
Grade 1 hypertension	637 (NA)	635 (34.5)	679 (23.7)	660 (14.4)	659 (11.8)	659 (10.3)
Grade 2 hypertension	558 (NA)	506 (11.7)	460 (7.4)	437 (5.9)	429 (5.1)	420 (4.8)
category unchanged,%		74.3	83.0	87.0	90.5	91.9
Cohens Kappa	NA	0.65 (0.63–0.68)	0.77 (0.75–0.79)	0.82 (0.80–0.84)	0.87 (0.85–0.89)	0.89 (0.87–0.90)

The absolute number of users in the BP- categories is shown at different time points of the HBPM protocol Classification is based on the cumulative BP mean of consecutive measurement days. The percentage of users who were reclassified compared to the previous day is given in brackets.

Analysis by BP classification tracking revealed an intensive exchange of users between directly neighbouring BP categories during the early to middle phase of the HBPM period, which continued at low level until the end of the HBPM period (Figure 3C). In contrast, more distant class switching occurred less often and early on (Figure 3C). Of note, a BP classified as normal after day 2 was never derived from a previous hypertensive reading and never switched to a hypertensive class, while a BP classified as grade I received significant contributions from the high normal class until the end of the measurement period (Figure 3C).

Since the greatest BP variability occurred in the early phase of HBPM, we examined the impact of excluding measurement day 1 on diagnosis and classification of hypertension. If the readings of the first day were omitted, the mean SBP (SD) decreased significantly from 131.7 (11.2) mmHg to 131.5 (11.3) mmHg (*p *< 0.0001 C.I. = 0.21–0.10). To investigate whether this change is clinically relevant, we analysed the relative change in the distribution of BD categories after exclusion of day 1. When the first day was omitted from HBPM, BP categories changed in 8.2% of cases (189 out of 2307). Different BP categories were affected to varying extent: while there was a 95.7% agreement in normal BP, accordance with full 6-day protocol in grade I hypertension was only 87.7% (Figure 3D). However, a more detailed analysis showed that excluding the first day was therapeutically relevant for a small proportion of patients. 8.5% of the patients initially classified as grade I HT were downgraded to high normal BP after omitting the first day (56 out of 659). In contrast, 4.1% of the users classified as high normal by a day 1–6 protocol were reclassified as grade I hypertension (32 out of 788). Overall, 3.8% of all users were affected by a therapeutically relevant reclassification after exclusion of day 1 (88 out of 2307, Figure 3D).

### 3.4. Absolute blood pressure values and hypertension control at baseline and during follow-up

To study the association of app usage and blood pressure change, we analysed BP values at baseline and 8–16 weeks after successful completion of the first HBPM period. Previous studies suggest that the BP lowering effect of app-based BP management tools can be detected after more than 8 weeks (22, 23). If a user performed more than one HBPM period within 8–16 weeks after completion of the first measurement week, the most recent HBPM period was defined as the follow-up period. Baseline characteristics of this subgroup (*n* = 864) are described in Table 3.

**Table 3:** Baseline characteristics of users with complete follow-up data (8-16 weeks).

Characteristics	Total (*N* = 864)	Female (*N* = 416)	Male (*N* = 448)
Age, years	62.2 ± 9.4	60.9 ± 8.7	63.5 ± 9.9
Age groups (%)			
18-30	1 (0.1)	1 (0.2)	0 (0.0)
31-40	13 (1.5)	5 (1.2)	8 (1.8)
41-50	79 (9.1)	46 (11.1)	33 (7.4)
51-60	269 (31.1)	150 (36.1)	119 (26.6)
61-70	333 (38.5)	154 (37.0)	179 (40.0)
> 71	169 (19.6)	60 (14.4)	109 (24.3)
BMI	27.4 ± 4.8	27.6 ± 5.3	27.2 ± 4.2
Systolic OBP, mmHg	137.4 ± 13.7 (*n* = 745)	138.2 ± 13.7	
(*n* = 369)	136.9 ± 13.8 (*n* = 376)		
Diastolic OBP, mmHg	83.1 ± 8.9 (*n* = 745)	83.1 ± 9.2 (*n* = 369)	82.5 ± 8.6 (*n* = 376)
OBP uncontrolled, %	50.2	51.2	49.2

Age, BMI, systolic and diastolic OBP values are expressed as mean ± SD. BMI, body mass index; OBP office blood pressure. Uncontrolled OBP: systolic BP ≥ 140 mm Hg and/or diastolic BP ≥ 90 mm Hg.

**Figure 4:**
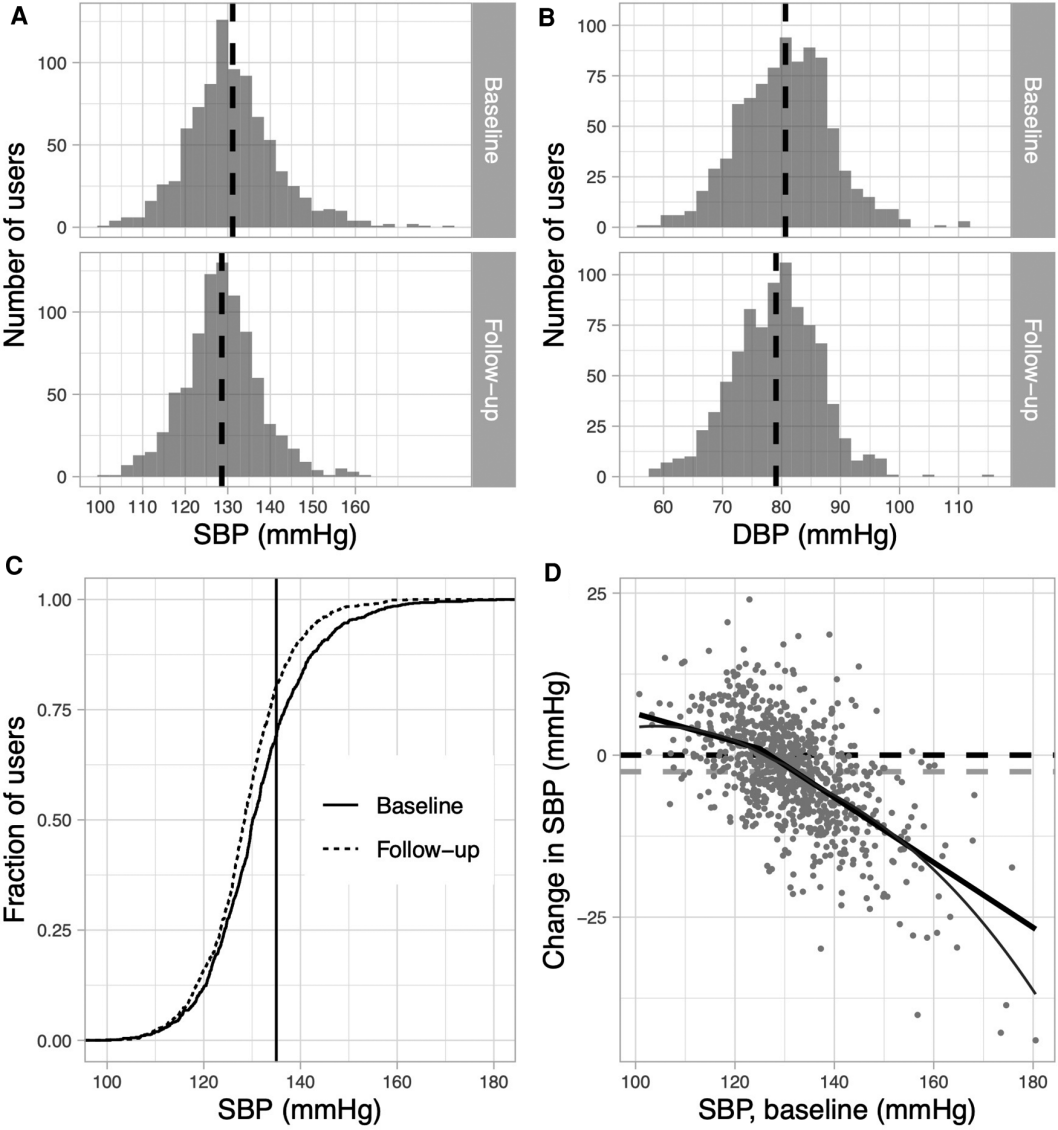
Blood pressure values and BP control at baseline and during follow-up. (A + B) Distribution of absolute systolic (**A**) and diastolic (**B**) BP- values at baseline and at follow-up. The dashed-line indicates the mean BP. (**C**) Fraction of patients by SBP (mean of entire HBPM period) at baseline (solid) and at follow-up (dashed). The vertical line indicates 135 mmHg; the threshold for diagnosing HT. (**D**) Change in SBP at follow-up according to baseline SBP: solid line indicates steady linear fitting of data. Thin grey line is a LOESS based guide-to-the-eye to visualise the non-linear relationship approximated by a piecewise linear model (bold solid line). The horizontal dashed line (grey) represents the average decrease in SBP. All analyses are based on data obtained from users with self-reported hypertension, who completed a HBPM-period at baseline and follow-up (*n* = 864).

Baseline mean (SD) SBP was 131.2 (10.8) mmHg, which was in the controlled range. After 8–16 weeks, the mean (SD) systolic blood pressure decreased significantly by 2.6 mmHg to 128.6 (8.9) mmHg (*p* < 0.0001, 95% C.I. = −3.09−2.02; Table 4, Figure 4A). Mean (SD) DBP decreased from 80.7 (7.8) at baseline to 79.1 (7.3) at follow up (*p* < 0.0001, 95% C.I. = −1.94−1.28; Figure 4B). In the subgroup of users with uncontrolled systolic BP (*n* = 384), mean (SD) SBP at baseline was 138.8 (9.9) mmHg and decreased significantly to 132.7(8.9) at follow up (*p *< 0.0001, 95% C.I. = −6.99−5.24; Table 4). Lower BP levels also resulted in changes in hypertension control. While at baseline, 55.6% of hypertensive users were controlled for systolic and diastolic BP, this proportion increased significantly to 68.1% at the follow-up HBPM period ([*X*^2^ (1, *N* = 864) = 10.92, *p* = .0009, Table 4]. Since the antihypertensive effects were most pronounced in users with high blood pressure, Pearson correlation coefficient was computed to assess the relationship between change in SBP and baseline SBP. Above a SBP of 125mmHg at baseline, there was a strong correlation with change in follow-up BP [*r*(624) = −.55, *p *< 0.0001], while below 125mmHg the degree of correlation was lower [*r*(236) =−.18, *p* = .004]. Participants with a very low baseline SBP showed a modest increase of blood pressure (Figure 4D).

**Table 4:** Blood pressure at baseline and follow-up of users with self-reported hypertension.

	Baseline	Follow-up (8–16 weeks)	*P* Value
*Absolute BP values*			
All hypertensive users (*n* = 864)	131.2 ± 10.8	128.6 ± 8.9	<0.0001
uncontrolled hypertensive users (*n* = 384)	138.8 ± 9.9	132.7 ± 8.9	<0.0001
*Hypertension control*			
Controlled BP in all hypertensive users, n (%)	480 (55.6%)	588 (68%)	0.0009

BP values are expressed as mean ± SD. Controlled BP <135/85 mmHg on average at home.

## 4. Discussion

With a digital HBPM-protocol, five consecutive measurement days are required for a reliable diagnosis and BP categorization. However, especially at therapeutic thresholds, additional measurement days are often required. Excluding day 1 from the protocol resulted in blood pressure reclassifications in 8.4% of all cases, with therapeutic relevance in 3.8% of cases.

By analysing data of app users with self-reported hypertension, we show that the use of a digital BP coach in a real-world setting is associated with a reduction in average BP and improvement of hypertension control after two periods of structured HBPM. Overall, the mean systolic BP decreased in all self-reported hypertensive users. The drop in BP was inversely correlated with baseline blood pressure (up to baseline BP > 125 mm Hg), therefore the decrease of BP was most pronounced in initially uncontrolled users. The reduction in absolute BP resulted in improved BP control. The clinical effects of such BP changes are difficult to estimate in a relatively uncharacterized user population with a normal average BP. Several studies have shown that the reduction in relative risk for all-cause mortality, but also for stroke or heart failure, is proportional to the extent of the BP decrease (24). Moreover, a recent meta-analysis was able to demonstrate a reduction in cardiovascular events even with normal baseline BP values. On average, a 5 mmHg reduction in blood pressure was associated with a 10% decrease in cardiovascular events (25). Therefore, it can be assumed that even small decreases in BP translate into clinical benefits. Since we only had access to self-reported data obtained in a non-controlled setting, the exact effect size of the app cannot be determined. In a recent randomised clinical trial investigating the effect of a smartphone coaching app in patients with uncontrolled hypertension, an 8.3 mmHg reduction in SBP was observed in the intervention group after 6 months (26). In this study, uncontrolled hypertensive users showed on average a BP reduction of 6.11 mmHg after 8–16 weeks. Various clinical studies have demonstrated that the effect of digital intervention/telemonitoring depends on the involvement of medical staff (26–28). Thus, the BP-lowering effect of the app could possibly be stronger if the intervention is formally integrated into a medical care system.

HBPM is recommended by guidelines for the diagnosis and management of hypertension (5, 7, 29). Despite this, only 51.0% of self-reported hypertensive patients in our study regularly performed a structured HBPM in the past. This shows the need for better support for patients with hypertension.

Guideline recommendations are inconsistent with regard to the design of the HBPM protocol (5, 7, 15). One important aspect of the HBPM protocol is the number of days required for appropriate diagnosis of hypertension. Several studies have investigated how many measurement days are required to determine true BP (17, 20, 30). Consistent with our findings, studies have proven that average BP decreases, as the total number of measurement days increases—with the largest changes occurring within the first 3 days (20). However, conclusions in current guidelines regarding the exact number of required days diverge (5, 7, 15). This may be due to the heterogeneity of the compared HBPM protocols as well as different methodological approaches in the various studies. The authors of a systematic review (based on 37 studies) suggested three days of HBPM in terms of the prognostic ability. Adding more measurement days did not result in further significant clinical benefit (20).

A recent study compared a protocol compatible with the current ESH recommendations with shorter versions (e.g., only 3 days). Interestingly, the mean blood pressure from 4.5 consecutive measurement days already showed good agreement with the standard protocol (31). We here performed a real-world data analysis based on a similar, guideline-compliant protocol, in which we stratified for BP categories, since this is highly relevant for prognosis and clinical management. We found that the time to reach a definitive BP category critically depends on baseline BP. Overall, the proportion of users who needed to be reclassified compared to the previous day dropped sharply from day 2–4 and was stable at <10% from day 5 onwards. However, intercategorial variation was high: users with grade II hypertension reached > 90% matching at day 2, while users with high-normal and grade I hypertension required at least 5 days. These data generally match results from the International Database on Home blood pressure in relation to Cardiovascular Outcomes (IDHOCO), which showed an 89.7% agreement at day 5 compared to the previous day. Moreover, the analysis from the IDHOCO dataset pointed out that reliability of BP-categorization depends on the initial blood pressure (17). We thus confirm these results in a real-world scenario.

Ideally, a HBPM protocol should be tailored to the individual case. In our analysis, there were pronounced class changes, especially in the area of therapeutically relevant threshold values (high normal BP, grade I hypertension). This was driven by bidirectional switching between directly neighbouring categories during the course of HBPM. Therefore, these users seem to require more measurement days for diagnostic certainty compared to users at the respective ends of the diagnostic spectrum. Of note, users with normal BP after day 2 never switch to a hypertensive class later (compared with the classification of the previous day).

In this context, exclusion of the first day is also often discussed, since a high blood pressure variability can be observed in the early phase of HBPM. However, in a recent meta-analysis exclusion of day 1 had no significant effect on the predictive value of HBPM (20). In line with this, in this study omitting day 1 resulted in only minor changes in classification overall. However, the most pronounced changes were seen in the subgroups close to therapeutic thresholds: grade I hypertension (12.3% reclassification) and high normal BP (8.0% reclassification). Overall, 3.8% of users were affected by a therapeutically relevant blood pressure class switch after omission of the first day. Even if those potentially therapeutically relevant changes only affect a small proportion of all users, it may have consequences on the individual level.

These results underline that general HBPM recommendations (in terms of required days, relevance of day 1) do not fully apply to all patients. In some cases, the clinician still needs to (re)adjust the protocol to obtain an adequate diagnosis. Therefore, the ideal HBPM protocol would need to be individually tailored to specific user requirements. This is an argument for an individual, patient-centred approach, especially in the context of digital applications. Individualised digital (algorithm based) HBPM could potentially improve both diagnostics and patient adherence.

Our study has limitations: The data are self-reported and were obtained in a non-controlled setting of voluntary and self-motivated users. The available data on demographic variables or antihypertensive medication are limited. Regarding the effect of the app, the user analysis therefore remains descriptive, further conclusions are not possible. This question requires a large, randomised, controlled trial (RCT). However, a recently published prospective RCT has already demonstrated a positive effect of an interactive app-based lifestyle change intervention. In a trial with 390 patients, the digital intervention resulted in a between-group difference of −4.3 mmHg in systolic home BP after 12 weeks (32). Moreover, in a setting of voluntary and self-motivated users, quality of recorded BP-values can only be estimated indirectly. It is possible that measurements were not always taken according to the current guideline recommendations. However, the digital coach motivates the user to carry out the BP-measurement correctly and regularly. Corresponding training modules are core elements of the app. Various studies have shown that HBPM provides highly accurate BP data, especially when compared to OBP (33). In contrast, OBP data sets are reported to be highly variable in both clinical practice and trials (34). Overall, the good agreement of our results with analyses based on large databases seems to imply a reasonable quality of the BP-data. However, analysing a real-world scenario offers the opportunity to examine the structure and function of HBPM in a context which patients and physicians are daily exposed to. Despite various limitations of real-world data, these datasets could help to transfer findings from controlled trials into a patient-centred digital context. Further in-depth studies on digital, individualised HBPM are required.

In conclusion, this analysis of real-world data suggests that HBPM provided by a digital coach may have the potential to improve diagnosis and therapy of home blood pressure. The use of the app is associated with a reduction in average BP and improvement of hypertension control. More importantly, this study confirmed that blood pressure categories on the population level were >90% accurate after 5 measurement days when implemented by a digital coach. However, the requirements for measurement days depend on the individual baseline BP and course and may need to be adjusted near the diagnostic threshold. For intelligent digital interventions, this diagnostic gap may offer the potential to further improve HBPM and facilitate diagnosis as well as monitoring of hypertension in future.

## Data Availability

The raw data supporting the conclusions of this article will be made available by the authors, without undue reservation.
