# Supplementary material for: Blood pressure dynamics during home blood pressure monitoring with a digital blood pressure coach—a prospective analysis of individual user data
Source: Front Cardiovasc Med. 2023 Apr 5;10:1115987. doi: 10.3389/fcvm.2023.1115987 (PMC10113611; doi:10.3389/fcvm.2023.1115987)
Supplement: Supplementary file 1 [file Datasheet1.docx]

Supplementary Material

**Blood pressure dynamics during Home Blood Pressure Monitoring with a digital Blood Pressure Coach - a prospective analysis of individual user data**

**Christian Beger^1,2^, Dominik Rüegger^3^, Anna Lenz^3^, Steffen Wagner^4,5^, Herrmann Haller^2^, Kai Martin Schmidt-Ott^2^, Dirk Volland^3^,** **Florian P. Limbourg^1,2*^**

*** Correspondence: Florian P. Limbourg**: Limbourg.Florian@mh-hannover.de

# Supplementary Tables

TABLE S1: systolic BP vs time (GAMM)

| R²_adj_ = 0.00113 | coefficient | *P* Value |
| --- | --- | --- |
| time | Smooth Curve, EDF = 1.00 | <2 x 10^-16^ |

TABLE S2: systolic BP vs time (linear model)

| R²_adj_ = 0.00113 | coefficient | *P* Value |
| --- | --- | --- |
| time | -0.246(33) | 2.14 x 10^-14^ |

TABLE S3: diastolic BP vs time (GAMM)

| R²_adj_= 0.000612 | coefficient | *P* Value |
| --- | --- | --- |
| time | Smooth Curve, EDF = 1.71 | 6.86 x 10^-8^ |

TABLE S4: diastolic BP vs time (linear model)

| R²_adj_ = 0. 000607 | coefficient | *P* Value |
| --- | --- | --- |
| time | -0.128(21) | 2.79 x 10^-10^ |

Table S5: systolic BP vs time and BP group (GAMM)

| R²_adj_ = 0.385 | coefficient | *P* Value |
| --- | --- | --- |
| Normal BP : time | Smooth Curve, EDF = 1.98 | <2 x 10^-16^ |
| High normal BP : time | Smooth Curve, EDF = 1.91 | 0.00541 |
| Grade I HTN : time | Smooth Curve, EDF = 1.91 | <2 x 10^-16^ |
| Grade II HTN : time | Smooth Curve, EDF = 1.98 | <2 x 10^-16^ |

# Supplementary Figures


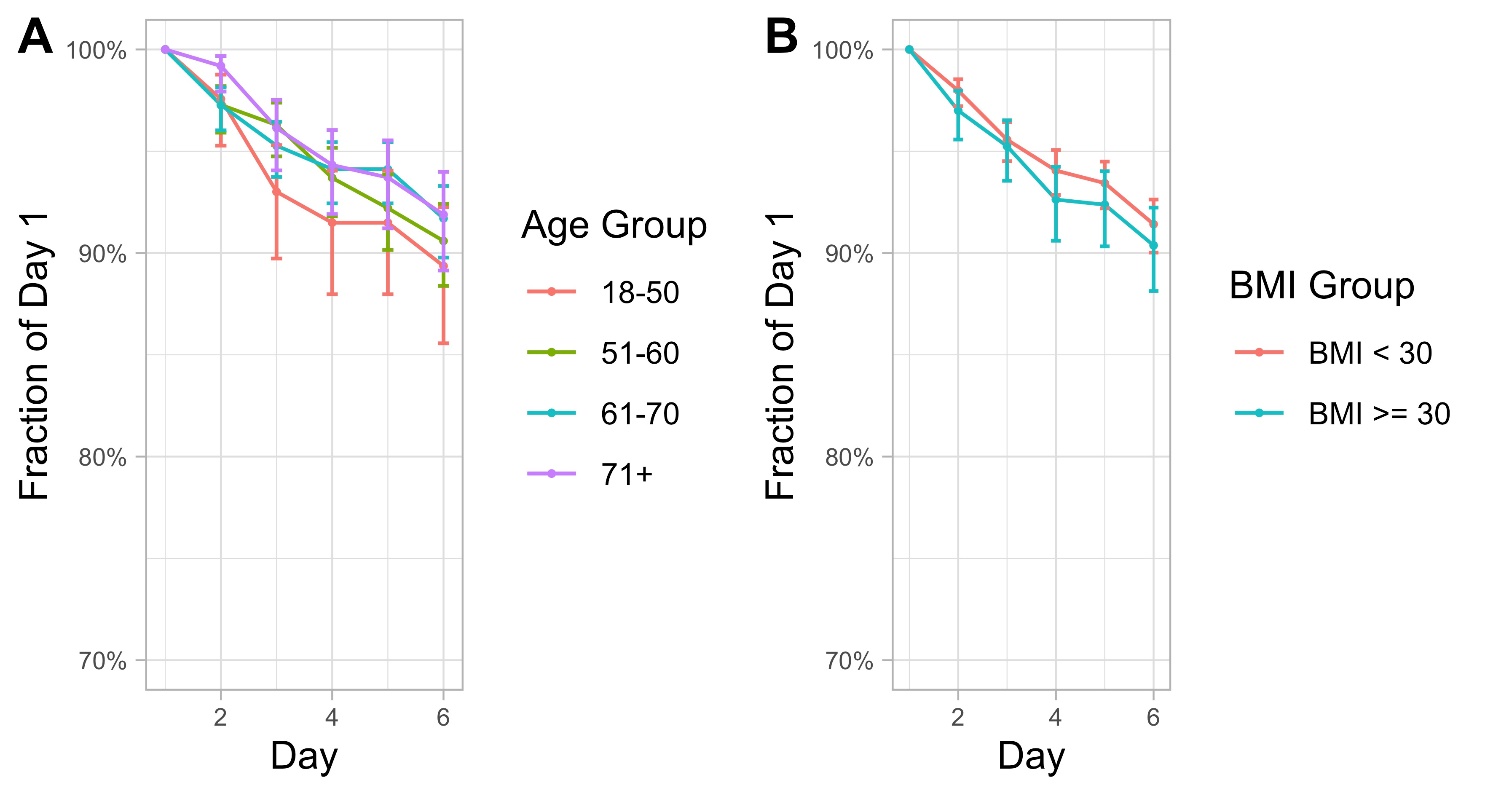


**Fig. S1 Adherence to the HBPM measurement protocol.** Development of user numbers during the first HBPM period, stratified by age group (A) and BMI (B). The percentage of users who completed measurements on a given day is displayed in relation to the total number of users on the first day of the HBPM period. Analyses are based on data from users with self-reported hypertension who enrolled in HBPM and completed the first day (n=2582).


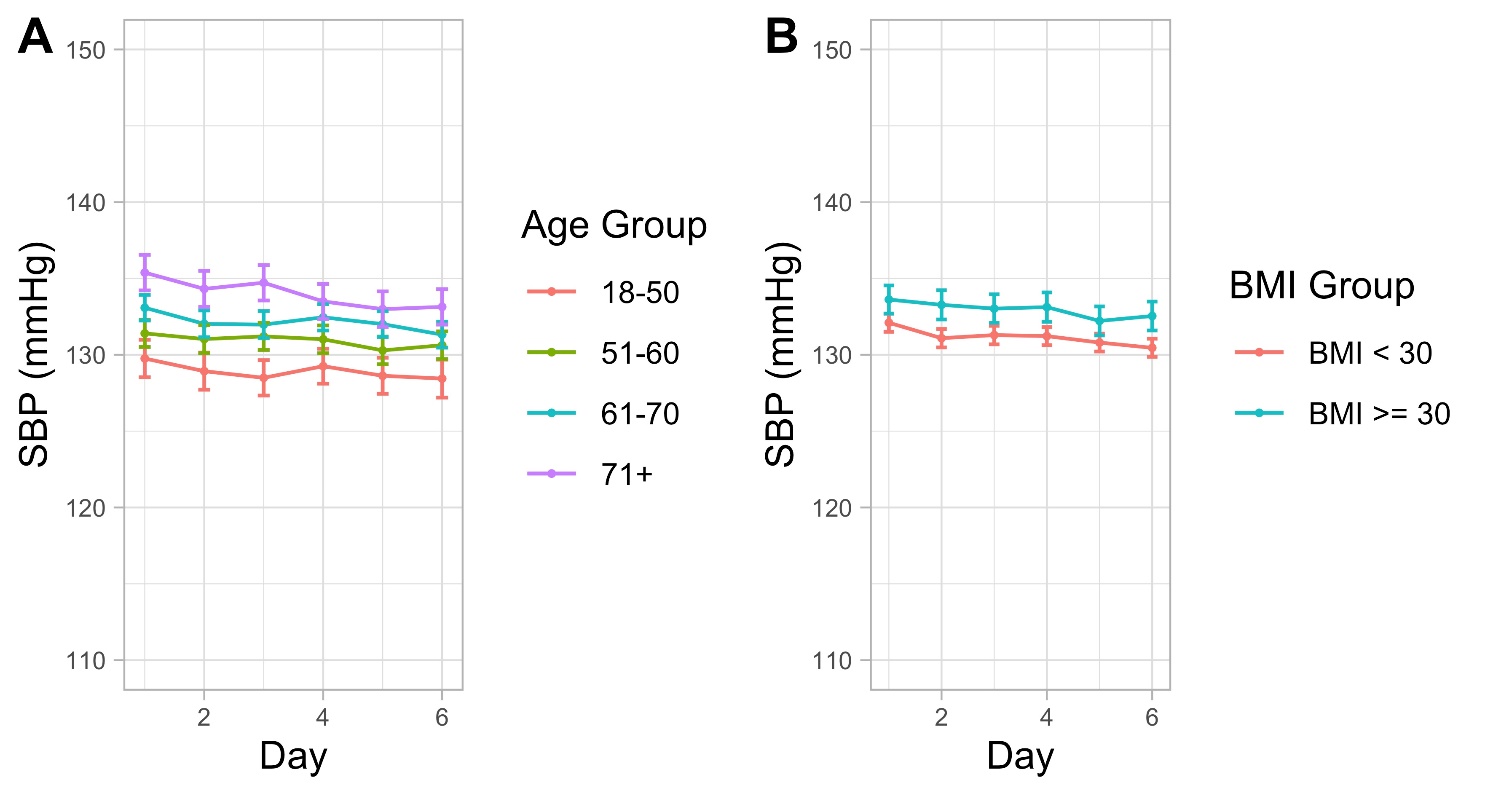


**Fig. S2** **Home blood pressure values ​​in the first HBPM interval.** Course of systolic blood pressure values of users with self-reported hypertension ​​in the first HBPM period stratified by age (A) and BMI (B). Error bars indicate 95% confidence interval. Analyses are based on data from users with self-reported hypertension who enrolled in HBPM and completed the first day (n=2582). BMI, body mass index; SBP systolic blood pressure.


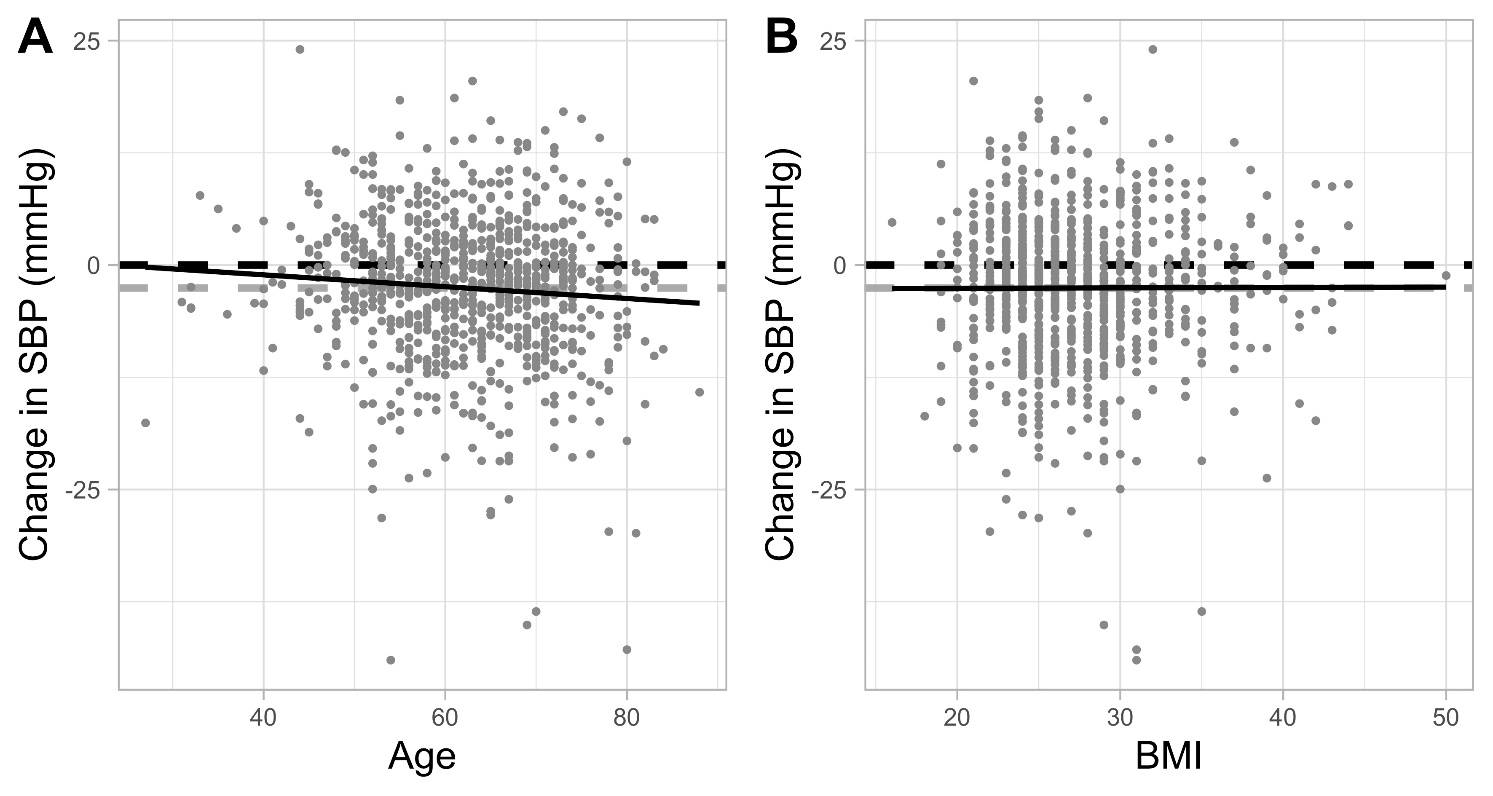


**Fig.** **S3 Change in systolic blood pressure at baseline and during follow-up.** Individual changes in SBP at follow-up according to age (A) and BMI (B). The horizontal dashed line (grey) represents the average decrease in SBP. The solid line indicates linear regression of data. All analyses are based on data obtained from users with self-reported hypertension, who completed a HBPM-period at baseline and follow-up (n=864). Pearson correlation coefficient was computed to assess the relationship between change in SBP (baseline vs follow-up) and age or BMI (age: r(862) = -.08, *p* 0.02; BMI: r(862) = -.00, *p* 0.94).
